# Drugs Cheaper Than Threepenny: The Market of Extremely Low-Priced Drugs within the National Health Insurance in Taiwan

**DOI:** 10.1155/2014/234941

**Published:** 2014-02-25

**Authors:** Bih-Ru Wang, Chia-Lin Chou, Chia-Chen Hsu, Yueh-Ching Chou, Tzeng-Ji Chen, Li-Fang Chou

**Affiliations:** ^1^Department of Pharmacy, Taipei Veterans General Hospital, Taipei 11217, Taiwan; ^2^Institute of Pharmacology, School of Medicine, National Yang-Ming University, Taipei 11217, Taiwan; ^3^College of Pharmacy, Taipei Medical University, Taipei 11031, Taiwan; ^4^Department of Family Medicine, Taipei Veterans General Hospital, Taipei 11217, Taiwan; ^5^Institute of Hospital and Health Care Administration, School of Medicine, National Yang-Ming University, Taipei 11217, Taiwan; ^6^Department of Public Finance, National Chengchi University, Taipei 11605, Taiwan

## Abstract

While most drug policy researches paid attention to the financial impact of expensive drugs, the market situation of low-priced drugs in a country was seldom analyzed. We used the nationally representative claims datasets to explore the status within the National Health Insurance (NHI) in Taiwan. In 2007, a total of 12,443 distinct drug items had been prescribed 853,250,147 times with total expenditure of 105,216,950,198 new Taiwan dollars (NTD). Among them, 7,366 oral drug items accounted for 701,353,383 prescribed items and 68,133,988,960 NTD. Besides, 2,887 items (39.2% of oral drug items) belonged to cheap drugs with the unit price ≤1 NTD (about 0.03 of US dollar). While the top one item among all oral drugs had already a market share of 5.0%, 30 items 30.3% and 107 items 50.0%, the cheap drugs with aggregate 332,893,462 prescribed items (47.5% of all prescribed oral drug items) only accounted for 2,750,725,433 NTD (4.0% of expenditure for oral drugs and 2.6% of total drug expenditure). The drug market of Taiwan's NHI was abundant in cheap drugs. The unreasonably low prices of drugs might not guarantee the quality of pharmaceutical care and the sustainability of a healthy pharmaceutical industry in the long run.

## 1. Introduction

The growth of drug expenditure is a global issue. Many governments have endeavored to control the drug expenditure in order to enhance the availability and affordability of drugs. The measures include product price control, reference pricing, and profit control [1–3]. Since its launch in 1995, the National Health Insurance (NHI) in Taiwan has offered a broad coverage of drug items, more than 20,000 items all the time [4]. Parallel to the growth of other sectors within the NHI, the drug expenditure has always accounted for one-fourth of the total NHI expenditure annually [5]. While most people pay attention to expensive drugs with the intention of cost control, a special phenomenon within the pharmaceutical sector of the NHI in Taiwan deserves careful study; the market is abundant in low-priced drug items. These drugs, mostly belonging to generics, might be the legacy of pharmaceutical regulations in earlier days of Taiwan. Perhaps the health insurance authorities intend to expel these drugs with old licences through a low-pricing strategy. However, the healthy development of an industry will depend on reasonable profits. The one-sided policy of setting low prices without persistent and extensive quality control might be detrimental to both the health of beneficiaries and the future of the pharmaceutical industry in Taiwan. A retrospective study is thus needed to observe the situation of low-priced drugs within the NHI before further discussions and measures can be undertaken.

In the current study, we analyzed the nationally representative claims datasets of the NHI in Taiwan to describe the market scale of drugs, with the special focus on those low-priced ones.

## 2. Materials and Methods

This study had been approved by the institutional review board of Taipei Veterans General Hospital, Taipei, Taiwan (2013-01-005E).

### 2.1. Data Sources

The data sources came from the National Health Insurance Research Database (NHIRD), which merged the NHI's electronic claims datasets into a large computerized database for research purpose [6, 7]. The single-payer NHI in Taiwan has covered almost all inhabitants (22,803,000 beneficiaries at the end of 2007, equaling 98.0% of all population) [8]. The database contains original claims for reimbursement in addition to registration files of beneficiaries and healthcare facilities. The identification numbers of persons and healthcare facilities in the datasets have been encrypted to protect privacy, but the encrypted identification numbers remain unique so that record-linking within datasets is feasible. All researchers who apply for use of the NHIRD are required to sign a written agreement declaring that they could not violate the privacy of patients or healthcare providers and should acknowledge the NHIRD on publication.

In the current study, we used three kinds of datasets as follows.

- The systematic sampling files of ambulatory care expenditures by visits (CD), details of ambulatory care orders (OO), inpatient expenditures by admissions (DD), and details of inpatient orders (DO): the datasets of CD and OO represent 1/500 of the original ambulatory claims datasets in each year and the datasets of DD and DO 1/20 of inpatient.
- The complete datasets of expenditures for prescriptions dispensed at contracted pharmacies (GD) and details of prescriptions dispensed at contracted pharmacies (GO): after increasing division of prescribing and dispensing and intensifying promotion of issuing the refill prescriptions for chronic illnesses within the NHI in recent years, the drug items dispensed outside the prescribing clinics, especially those in the second and third refills, can only be known from GD and GO datasets. The GD and GO datasets are indispensable complements to the prescribing datasets of CD and OO.
- The registry for drug items: the Bureau of NHI releases the registry of reimbursable drug items on its web site (http://www.nhi.gov.tw) monthly. The registry is cumulative; that is, it includes the historical pricing details of each drug item.

### 2.2. Study Design

For favorable comparisons, the focus was on oral drugs with solid forms, that is, tablets and capsules. A cheap drug was defined as being with a unit price ≤1 new Taiwan dollar (NTD), about 0.03 US dollar.

For each distinct drug item, we calculated how many times it had been prescribed in the whole year of 2007. The aggregate prescribed pill count and drug cost were also calculated. The data from the systematic sampling OO datasets were multiplied with 500 in case of dispensing at clinics and the data from the systematic sampling DO datasets were multiplied with 20. They were then merged with the data from the complete GO datasets representing the drugs dispensed at outside pharmacies. Because unit prices of drugs might change during the year, we adopted the last approved price of each drug item at the end of 2007 for data grouping by drug unit price.

The analyses were stratified by drug item and unit price. The market share referred to the percentage of drug costs attributed to a certain drug item or a group of drug items in all drug costs.

### 2.3. Data Processing and Statistical Analysis

The open-source software Perl (version 5.18.0) was used for computing. The regular statistics were displayed.

## 3. Results

**Figure 1:**
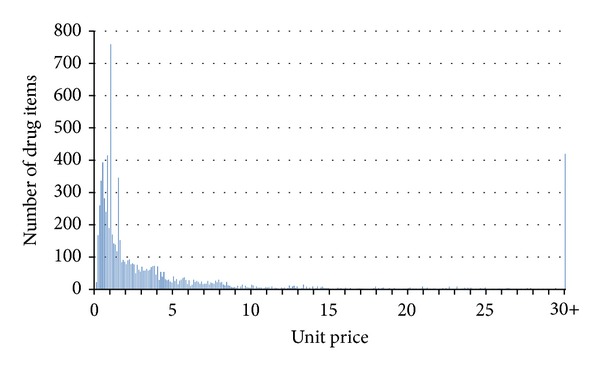
Unit price distribution of oral pills (tablets and capsules) reimbursable within Taiwan's National Health Insurance in 2007.

According to the master file of drugs reimbursed by the NHI, 8,060 items of all 15,870 drug items in 2007 were in solid form for oral intake. Among all the oral pills, nearly one-fourth of drug items are with a unit price between 0.5 and 1 NTD and totally 38.0% of drug items are cheaper than 1 NTD (Table 1). Two drug items of chlorpheniramine, a conventional antihistamine, are the cheapest with a unit price of 0.06 NTD for one tablet of 4 mg.  Figure 1 shows the unit price distribution of oral pills reimbursable within Taiwan's NHI in 2007.

In 2007, a total of 12,443 distinct drug items had been prescribed 853,250,147 times with total expenditure of 105,216,950,198 NTD. Among them, 7,366 oral drug items accounted for 701,353,383 prescribed items (82.2% of total prescribed items) and 68,133,988,960 NTD (64.8% of total drug expenditure). A total of 694 reimbursable oral drug items had been never prescribed in the year. According to the last approved price of each prescribed oral drug item before the end of 2007, the average unit price was 10.0 ± 95.1 NTD, with minimum 0.06, maximum 4,270, and medians 1.5, 25 percentile 0.8 and 75 percentile 4.31 and 2,887 items (39.2% of prescribed oral drug items) had a unit price ≤1 NTD.

**Figure 2:**
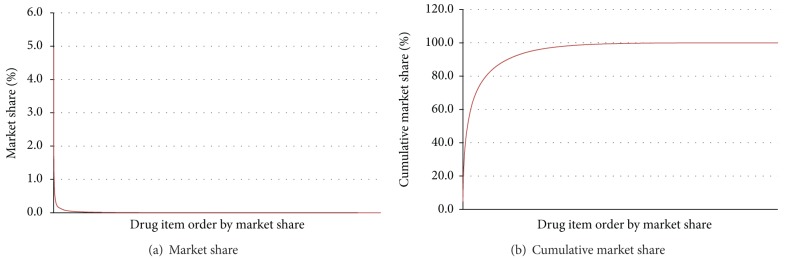
Market share of oral pills (tablets and capsules) prescribed within Taiwan's National Health Insurance in 2007. (a) Market share. (b) Cumulative market share.

When the market share of each oral drug item in terms of cost was ranked, the top one drug item with a unit price of 18.1 NTD (Norvasc [amlodipine] 5 mg) had already a market share of 5.0% (Table 2). The top 30 items accounted for 30.3% of the market and the top 107 accounted for 50.0%. On the other hand, 4,441 drug items accounted for only 1.0% of the market and 6,327 items accounted for 10.0%.  Figure 2 shows the market share and cumulative market share of oral pills prescribed within Taiwan's NHI in 2007.

For the cheap drug items, that is, those with a unit price not greater than 1 NTD, they had been aggregately prescribed 332,893,462 times (47.5% of all prescribed oral drug items) with a pill count of 3,905,197,879 (32.7% of all prescribed oral pills) but only accounted for 2,750,725,433 NTD (4.0% of expenditure for oral drugs and 2.6% of total drug expenditure) (Table 3).

## 4. Discussion

Our current study revealed that low-priced drug items abounded in the pharmaceutical market within the NHI in Taiwan. These low-priced drugs, possibly belonging to generics, were highly frequently prescribed, but their market share in terms of drug cost was very modest. The phenomenon might face a dilemma: to contain the drug costs without doing any harm to the sustainability of the pharmaceutical market.

Most countries in the world, both developed and developing, have the same problem in containing the drug cost [1–3]. The promotion of generic drugs (“generic substitution”) is one of the major measures with the intention of introducing more price competition [9–11]. In recent years, it has been even proposed in the USA to import more generic drugs from other countries or, in other words, to deem the international pharmaceutical market as a source of low-priced prescription drugs [12]. Among the developed countries, Cyprus, a small island country in the Mediterranean Sea, is a special example [13]. Due to its small population and small indigenous pharmaceutical industry, pharmaceuticals are mainly imported. The regulations about drug prices in Cyprus created perverse incentives for wholesalers to import expensive products, leading Cyprus to a country with high-price drugs.

Works about generic drugs in developing countries have a long tradition fostered by the World Health Organization. In recent years, the World Health Organization has closely cooperated with the Health Action International (HAI), a Dutch nongovernment organization, to make existing drug price information more widely available in order to improve equity in access to essential medicines in health systems of developing and middle-income countries [14, 15]. In addition to cost containment, the affordability and availability are the main foci of concerns.

Within the NHI in Taiwan, the prices of reimbursed drugs are in most of the cases unilaterally set by the Bureau of National Health Insurance through an in-house team, an expert committee, and a series of formal procedures [16, 17]. Although extensive surveys of drug market prices have performed five rounds and are accompanied by cutting back on reimbursement prices of many drugs in the past [18], the annual drug expenditure within the NHI still increased to 125 billion NTD in 2008 [19]. Therefore, a more drastic and extensive reduction of drug prices was undertaken by the Bureau of National Health Insurance in 2009, which was believed to have profound influences on the cost structures both within the health care facilities and within the pharmaceutical sector. The impact on the evolution of low-priced drugs deserves attention.

Reflections on the abundance of low-priced drugs within the NHI in Taiwan included whether the reimbursement of such drugs was worthy. It could be imagined that the cost of prescribing and dispensing would far exceed that of drugs per se. However, further analyses were needed to illustrate the recipients of low-priced drugs and the indications for their prescriptions.

## 5. Conclusions

The drug market of the National Health Insurance in Taiwan was abundant in cheap drugs. The unreasonably low prices of drugs might not guarantee the quality of pharmaceutical care and the sustainability of a healthy pharmaceutical industry in the long run.

## Figures and Tables

**Table 1:** Oral pills (tablets and capsules) reimbursable within Taiwan's National Health Insurance in 2007.

Range of drug unit price in NTD^a^	Number of items	Percentage	Cumultive number of items	Cumultive percentage
≤0.1	22	0.3%	22	0.3%
(0.1, 0.2]	168	2.1%	190	2.4%
(0.2, 0.3]	260	3.2%	450	5.6%
(0.3, 0.4]	336	4.2%	786	9.8%
(0.4, 0.5]	393	4.9%	1,179	14.6%
(0.5, 1.0]	1,886	23.4%	3,065	38.0%
(1.0, 2.0]	1,404	17.4%	4,469	55.4%
(2.0, 3.0]	730	9.1%	5,199	64.5%
(3.0, 5.0]	979	12.1%	6,178	76.7%
(5.0, 10.0]	895	11.1%	7,073	87.8%
(10.0, 20.0]	382	4.7%	7,455	92.5%
(20.0, 30.0]	189	2.3%	7,644	94.8%
>30.0	416	5.2%	8,060	100.0%

Total	8,060	100.0%		

NTD: new Taiwan dollar.

^a^(*x*, *y*] that is denoted as the price is above *x* and equal to or less than *y*.

**Table 2:** Market share of costs by drug item within Taiwan's National Health Insurance in 2007.

Drug*	Number of items	Market share	Cumultive market share
1	1	5.0%	5.0%
2–4	3	5.1%	10.0%
5–13	9	10.1%	20.1%
14–30	17	10.2%	30.3%
31–58	28	9.8%	40.1%
59–107	49	9.9%	50.0%
108–182	75	10.1%	60.1%
183–306	124	9.9%	70.0%
307–540	234	10.0%	80.0%
541–1039	499	10.0%	90.0%
1040–1588	549	5.0%	95.0%
1589–2925	1,337	4.0%	99.0%
2926–7366	4,441	1.0%	100.0%

*Focus was on oral drugs with solid forms, that is, tablets and capsules.

**Table 3:** Utilization pattern of oral pills (tablets and capsules) within Taiwan's National Health Insurance in 2007, stratified by unit price.

Drug unitprice^a^	Number of items	Prescribed frequency		Prescribed pill amount		Drug cost	
		Number (%)	Cumultive percentage	Number (%)	Cumultive percentage	Cost (NTD) (%)	Cumultive percentage
≤1	2887	332,893,462 (47.5)	47.5%	3,905,197,879 (32.7)	32.7%	2,750,725,433 (4.0)	4.0%
(1-2]	1291	137,680,995 (19.6)	67.1%	2,748,567,233 (23.0)	55.8%	4,353,528,777 (6.4)	10.4%
(2-3]	644	55,141,275 (7.9)	75.0%	1,002,985,634 (8.4)	64.2%	2,811,873,496 (4.1)	14.6%
(3–5]	906	69,862,237 (10.0)	84.9%	1,402,193,271 (11.8)	76.0%	6,344,373,650 (9.3)	23.9%
(5–10]	786	49,528,347 (7.1)	92.0%	1,388,364,820 (11.6)	87.6%	10,943,790,384 (16.1)	39.9%
(10–20]	343	25,795,894 (3.7)	95.7%	705,197,333 (5.9)	93.5%	10,994,223,793 (16.1)	56.1%
(20–30]	169	14,720,103 (2.1)	97.8%	400,773,824 (3.4)	96.9%	9,579,571,005 (14.1)	70.1%
>30	340	15,731,070 (2.2)	100.0%	374,096,181 (3.1)	100.0%	20,355,902,421 (29.9)	100.0%

Total	7366	701,353,383 (100.0)		11,927,376,174 (100.0)		68,133,988,960 (100.0)	

NTD: new Taiwan dollar.

^a^(*x*, *y*] that is denoted as the price is above *x* and equal or less than *y*.
